# Understanding the Developmental Pathways and Onset of Bipolar Disorder and Borderline Personality Disorder in Young People: A Systematic Review of Reviews

**DOI:** 10.1192/bjo.2022.189

**Published:** 2022-06-20

**Authors:** Buse Beril Durdurak, Rachel Upthegrove, Nada Altaweel, Steven Marwaha

**Affiliations:** University of Birmingham, Birmingham, United Kingdom

## Abstract

**Aims:**

There is still an ongoing debate on the nosological position of Bipolar Disorder (BD) and Borderline Personality Disorder (BPD). Identifying the unique and shared risks and developmental pathways in emerging BD and BPD could help the field refine aetiological hypotheses of these disorders. The study aims were to systematically synthesise the available evidence from systematic reviews and meta-analyses concerning environmental, psychosocial, biological, and clinical factors leading to the emergence of BD and BPD to identify the main differences and common characteristics between the two disorders to characterise their complex interplay whilst highlighting remaining evidence gaps.

**Methods:**

A literature search was conducted PubMed, PsychINFO, EMBASE, Cochrane, CINAHL, MEDLINE, and ISI Web of Science as the data sources. 19 systematic reviews and meta-analyses involving 217 prospective studies met eligibility criteria.

**Results:**

Results demonstrated that family history of psychopathology, affective instability, attention deficit hyperactivity disorder, anxiety disorders, depression, sleep disturbances, substance abuse, psychotic symptoms, suicidality, childhood adversity and temperament dimensions were common predisposing factors across both disorders. There are also many distinct variables that could be found early in the course of both disorders. Most of the factors should be considered as a general, nonspecific precursor signs and symptoms of both BPD and BD, apart from subsyndromal depression, subsyndromal hypomania, cyclothymia disorder, psychotic symptoms, age at onset of major depression and frequency and loading of affective symptoms.

**Conclusion:**

Although the findings of this review may lead to support the view of BD and BPD as two distinct disorders, there is not sufficient data to either indicate that BD and BPD are separate nosological entities or that BPD should be considered as an extension of BD disorders. Future research is required to increase our understanding of the aetiology of BD and BPD onset and their complex interplay by conducting prospective studies which concurrently examine multiple measures including biological, environmental, psychosocial and clinical factors in BD and BPD at-risk populations. Large, multilevel data sets will enable deep phenotyping and distinguish pathophysiological pathways.

